# Evaluation of N-NOSE as a surveillance tool for recurrence in gastric and esophageal cancers: a prospective cohort study

**DOI:** 10.1186/s12885-024-13327-x

**Published:** 2024-12-18

**Authors:** Sayuri Iitaka, Akihiro Kuroda, Tomonori Narita, Hideyuki Hatakeyama, Masayo Morishita, Umbhorn Ungkulpasvich, Takaaki Hirotsu, Eric di Luccio, Koichi Yagi, Yasuyuki Seto

**Affiliations:** 1 Department of Gastrointestinal Surgery, Graduate School of Medicine, The University of Tokyo, Tokyo, 113-8655 Japan; 2 Hirotsu Bio Science Inc, Tokyo, 102-0094 Japan; 3 Present address: National Cancer Center Hospital, Tokyo, 104-0045 Japan

**Keywords:** *Caenorhabditis elegans* (*C. elegans*), Urine, Nematode-nose (N-NOSE), Gastric cancer, Oesophageal cancer

## Abstract

**Objective:**

Early detection of recurrent gastric and esophageal cancers remains a critical challenge. Innovative and non-invasive cancer screening technologies, such as N-NOSE, can improve early detection. N-NOSE is a urine-based scent test that leverages the olfactory abilities of the nematode *C. elegans*. For the first time, this prospective study evaluates the efficacy of the N-NOSE chemotaxis index as a novel biomarker for postoperative surveillance and recurrence in patients with upper gastrointestinal cancers.

**Methods:**

A two-year prospective cohort study was conducted at The University of Tokyo Hospital, involving 40 patients with gastric and esophageal cancers. Urine samples were collected pre- and postoperatively and analysed using the N-NOSE technique.

**Results:**

In cases of recurrence with vascular invasion, the chemotaxis index at 100-fold urine dilution was significantly elevated compared to the non-recurrence group.

**Conclusion:**

This study suggests the potential of N-NOSE as an effective follow-up tool for patients with upper gastrointestinal cancer, particularly those with vascular invasion. While N-NOSE has been validated to distinguish between cancer and non-cancer, and its performance compared to traditional markers has been proven, it has not been studied for recurrence. Our data highlights, for the first time, the capability of N-NOSE in the surveillance of cancer recurrence.

**Supplementary Information:**

The online version contains supplementary material available at 10.1186/s12885-024-13327-x.

## Background

Gastric cancer still accounts for 1 million new cases per year worldwide and is the fourth leading cause of cancer death. The incidence is higher in East Asia and Eastern Europe. Oesophageal cancer accounts for 600,000 new cases per year and is the sixth leading cause of cancer death [1].

Postoperative surveillance is a critical component of patient care following surgical intervention for gastric cancer. The primary objective of this follow-up care is to facilitate the early detection of recurrent disease and the identification of secondary malignancies. Despite the importance of this process, robust evidence is needed to guide the selection of appropriate diagnostic modalities and the determination of optimal surveillance intervals.

In the context of oesophageal cancer, the recurrence rate following curative resection is alarmingly high, reported to be between 40–60%. Approximately 80–90% of these recurrences manifest early, typically within the first two years following surgery. The prognosis for patients with recurrent disease is generally poor, underscoring the urgency of early detection and intervention.

The ability to detect recurrence at an early stage can significantly alter the clinical trajectory of these patients. Early therapeutic intervention, facilitated by vigilant postoperative surveillance, can improve survival outcomes. Therefore, rigorous postoperative surveillance must be considered [2–6].

While tumor markers and imaging are commonly used for the surveillance of gastric and oesophageal cancers, their limitations in early recurrence detection underscore the need for more reliable methods. Recently, there has been a surge of interest in novel biological diagnostics, such as circulating tumor DNA (ctDNA), liquid biopsies, olfaction-based approaches, and epigenetic markers. These emerging technologies offer the potential for non-invasive, highly sensitive detection of tumor recurrence. Integrating biological diagnostics into postoperative surveillance could significantly transform the management of gastric and oesophageal cancers, facilitating early intervention and improving patient outcomes [7–12].

Nematode-based tests utilizing *Caenorhabditis elegans* (N-NOSE) are an emerging approach for non-invasive early cancer detection, leveraging the nematode's highly attuned olfactory system. *C. elegans* has shown promise in detecting cancer-specific volatile organic compounds (VOCs) in bodily fluids, exhibiting distinct chemotactic responses to samples from cancer patients compared to healthy controls. Specifically, C*. elegans* is attracted to the urine of cancer patients while avoiding that of healthy individuals. N-NOSE, which applies the nematode's sense of smell, demonstrated high accuracy with a sensitivity of 95.8% and specificity of 95.0%, as reported by Hirotsu et al. in their study involving 24 cancer patients and 218 healthy subjects [13]. Kusumoto et al. investigated the diagnostic performance of N-NOSE in 180 gastrointestinal cancers and 76 healthy subjects and found that N-NOSE showed an AUC of 0.80 or higher regardless of cancer stage. The AUC of N-NOSE was significantly higher than that of CEA and CA19-9 in diagnosing gastric and oesophageal cancer [14].

However, there are few reports on N-NOSE's clinical performance for gastroesophageal cancer detection [13–17] and only limited data on changes before and after surgery [15, 17]. Additionally, reports on using N-NOSE for recurrence surveillance are scarce, with only three cases of recurrence documented [15]. Therefore, this study aims to examine whether the N-NOSE chemotaxis index varies depending on disease recurrence. Furthermore, this study evaluates whether the N-NOSE diagnostic method can serve as a biomarker for follow-up after surgery.

## Material and methods

### Study populations

Patients with upper gastrointestinal cancer scheduled for radical surgery at the Department of Gastroesophageal Surgery, The University of Tokyo Hospital, between October 2020 and December 2021 were enrolled.

Eligible patients were those who were at least 20 years old at the time of enrolment, had been histologically diagnosed with gastric or oesophageal cancer, whose Eastern Cooperative Oncology Group (ECOG) Performance status (PS) of 0 or 1, had preserved organ function, and who were planned for radical resection. Patients with synchronous or metachronous (within five years) cancers of other organs, except for intraepithelial or intramucosal carcinoma cured by local therapy and active systemic infectious disease, were excluded from this study.

### Treatment strategy and collection of urine samples

**Fig. 1:**
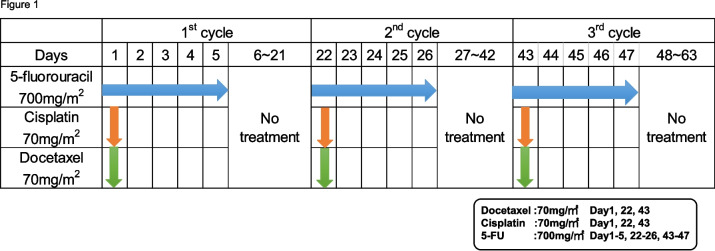
Preoperative chemotherapy regimen. 10 patients in this study received chemotherapy in the method of the chemotherapy regimen that repeated 3 times the 21-day cycle of DCF medicine administration schedule. Docetaxel (70 mg/m^2^), Cisplatin (70 mg/m^2^) and 5-fluorouracil (5-FU, 700 mg/m^2^) were administered on the first day of the cycle and only 5-FU was continued every day to 5th day of the cycle. Then the rest 16 days (6th −21th day) had no medicine administration

Preoperative docetaxel, cisplatin, 5-FU (DCF) therapy is considered standard treatment for resectable locally advanced oesophageal cancer in Japan [18, 19]. In this study, cases classified as Stage II/III (excluding T4) according to the TNM classification (UICC 8th edition) underwent preoperative treatment with docetaxel (70 mg/m^2^) + cisplatin (70 mg/m^2^) + 5-FU (700 mg/m^2^) (DCF) therapy for three courses (Fig. 1).

Staging was based on the TNM classification (UICC 8th edition) [20]. Measurable lesions were evaluated with computed tomography (CT). They were assessed in accordance with the Response Evaluation Criteria in Solid Tumors Criteria (RECIST) version 1.1 [21]. Recurrence was diagnosed by CT or positron emission tomography-computed tomography (PET-CT) during postoperative surveillance.

**Fig. 2:**
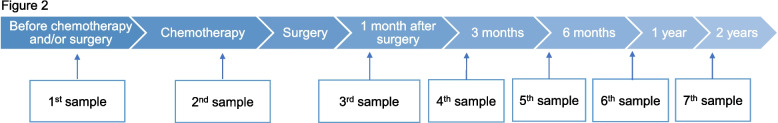
Schematics of urine sampling. Urine samples were collected 6 times, before chemotherapy and/or surgery, at 1 month, 3 months, 6 months, 1 year, and 2 years after surgery. Only urine samples of esophageal cancer patients who underwent preoperative chemotherapy were additionally collected right after the completion of preoperative chemotherapy

Urine samples were collected six times before chemotherapy and/or surgery, and at one month, three months, six months, one year, and two years after surgery. Urine samples were collected during fasting. For esophageal cancer patients who underwent preoperative chemotherapy, urine samples were collected just after preoperative chemotherapy (Fig. 2). Collected urine samples were rapidly frozen at below −20℃ within two hours of sampling and remained frozen until use for N-NOSE.

### Culture and maintenance of *C. elegans*

*C. elegans* (wild-type N2) was cultured with standard methods at 20 °C on Nematode Growth Media (NGM) seeded with *Escherichia coli* (*E. coli*) strain NA22 as a food source [13].

### Measurement method of N-NOSE

N-NOSE is a technique based on the olfactory response of the nematode *C. elegans*, which displays an attractive chemotaxis toward cancer patients’ urine, whereas it shows an aversive response to non-cancerous ones [22, 23]. The chemotaxis analysis was performed according to the standard protocols used in previous nematode studies [13].

*C. elegans* (wild-type N2) was cultured at 20 °C under well-fed and uncrowded conditions with the *E. coli* strain NA22 as a food source. Chemotaxis assays were performed on 9-cm agar plates containing 10 mL of 2% agar, 5 mM KPO_4_, 1 mM CaCl_2_, and 1 mM MgSO_4_ [14]. Briefly, 0.5 μL of 1 M sodium azide was spotted on two points at both ends of the plates. Sodium azide is the anesthetic used to minimize the effects of adaptation. One μL of urine sample was added to two points at one side of the plate, leaving the other side with sodium azide alone. Following the addition of sodium azide and urine sample, approximately 100 adult nematodes that were collected and washed three times with chemotaxis buffer (0.05% gelatin, 5 mM KPO_4_, 1 mM CaCl_2_, and 1 mM MgSO_4_), were placed in the center of the plate. After removing the excess buffer, the nematodes were allowed to roam for 30 min. The chemotaxis index was calculated using the following equation: Chemotaxis index = (A—B)/(A + B), where (A) is the number of nematodes near the urine samples, and (B) is the number of nematodes in the region without the urine samples. For each sample, the chemotaxis assays were performed using urine samples at 2 different dilutions, tenfold or 100-fold (*n* = 10 for each dilution). A negative index (−1 to 0) indicates repulsion to the sample, and a positive index (0 to 1) indicates an attraction to the sample. Throughout this study, the nematode chemotaxis experiment was strictly conducted with no information on the clinical manifestation and therapeutic progress of the samples analyzed.

### Statistical analyses

The statistical analyses used a t-test to compare continuous variables between the two groups. ROC analyses based on logistic regression were also performed. Statistical significance was defined as a *p*-value of less than 0.05. JMP Pro® ver. 17.2 (SAS Institute, Cary, NC, USA) was used.

## Results

**Fig. 3:**
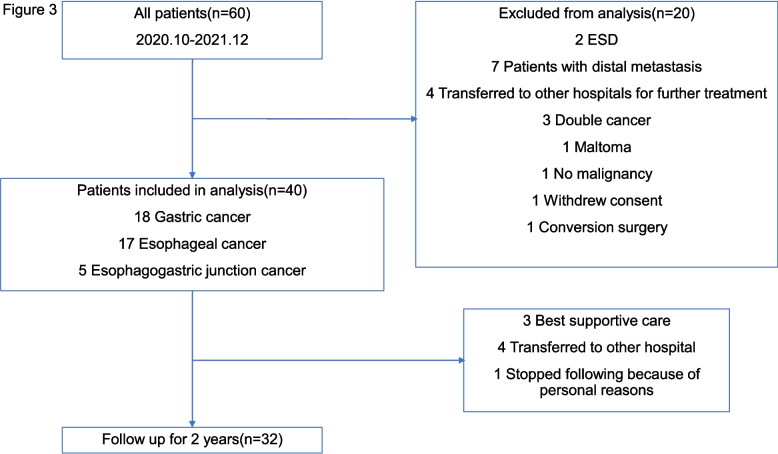
The chart of exclusion criteria in this study. Sixty patients were initially enrolled in this study; 20 were excluded based on exclusion criteria. The remaining 40 patients included. Of these, 32 patients were followed up for 2 years after surgery

To evaluate whether the N-NOSE technique can serve as a biomarker for upper gastrointestinal cancer recurrence and postoperative surveillance, *C. elegans* chemotaxis assays and statistical analyses were performed using urine samples from cancer patients. Sixty patients were initially enrolled in this study; 20 were excluded based on exclusion criteria (Fig. 3). The remaining 40 patients included 18 with gastric cancer, 17 with esophageal cancer, and 5 with esophagogastric junction cancer, all of whom underwent the N-NOSE cancer test. Of these, 32 patients were followed up for 2 years after surgery (Fig. 3). Patient characteristics and cancer stage information are detailed in Table 1. The median age was 71 years (range: 27–91), with 30 males and 10 females.

**Table 1:** Characteristics of the patients in this study

Characteristics	Gastric cancer (*N* = 18)	Esophageal cancer (*N* = 17)	Esophagogastric junction cancer (*N* = 5)	Total (*N* = 40)
Age(years)				
Median	73	69	80	71
Range	27–91	49–85	60–82	27–91
Gender				
Female	6	2	2	10
Male	12	15	3	30
Stage				
I	11	7	0	18
II	3	4	1	8
III	4	5	4	13
IV	0	1	0	1
Pathological T stage				
1	8	8	0	16
2	3	4	1	8
3	4	4	4	12
4a	3	1	0	4
Pathological N stage				
0	13	10	1	24
1	1	2	3	6
2	2	4	1	7
3	2	1	0	3

Urine samples were collected from each patient multiple times before and after surgery as described in Materials and Methods, with a schematic of sampling shown in Fig. 2. The chemotactic behavior and odor preference of *C. elegans* depend on odor concentrations [22, 24]. Based on this, N-NOSE accuracy evaluation requires two urine concentrations [16]. Therefore, 10- and 100-fold dilutions were used in this study. Chemotaxis assays were performed using urine samples from all 40 patients, with 10 repetitions per sample (*n* = 10).

Bar graphs in Supplemental Fig. 1 show the averaged chemotaxis indexes (*n* = 10) of 100-fold diluted urine samples, ranked from highest to lowest (samples numbered as 1 to 40) before chemotherapy and/or surgery (Supplemental Fig. 1A). Chemotaxis indexes for urine collected at after chemotherapy, 1, 3, 6 months, 1 year, and 2 years after surgery are shown in Supplemental Figs. 1B-G. 34 patients urine samples resulted in a positive chemotaxis index in the N-NOSE test and showed a reduced chemotaxis index from preoperative to postoperative samples (Supplemental Fig. 1A-C). Patients #28–33 resulted in a negative chemotaxis index for the first sample remained negative index through the observation except patient #31 (Supplemental Fig. 1A-G). In recurrent cases, all patients (#34–40) resulted in a positive chemotaxis index (attraction) for the first sample in 100-fold diluted urine compared to tenfold dilution (Supplemental Fig. 2). Except patient #40, an increasing trend in the chemotaxis index was observed to 1-year after surgery (Supplemental Figs. 1C-F). At the 2-year follow-up, patients #34, 36, 38, 39 showed a reduction in the chemotaxis index (Supplemental Fig. 1G).

### Post-surgery outcomes: recurrence and non-recurrence cases

We examined whether the chemotaxis index was associated with recurrence. When comparing the recurrence group and the non-recurrence group, the chemotaxis indexes (1st sample) were not significantly greater in the recurrence group than in the non-recurrence group (Table 2).

**Table 2:** Comparison of recurrence group and non-recurrence group

Chemotaxis index	Non-recurrence	Recurrence	*P*-value
(1st sample)	*n* = 33	*n* = 7	
Median (10-dilution)	0.092	0.07	0.776
[interquartile range]	[0.053, 0.130]	[0.012, 0.254]	
Median (100-dilution)	0.056	0.084	0.474
[interquartile range]	[0.025, 0.100]	[0.084, 0.117]	

Analysis using chemotaxis indexes of urine samples of 40 cases including 7 recurrence and 33 non-recurrence patients did not show a significant association of chemotaxis index and recurrence

**Fig. 4:**
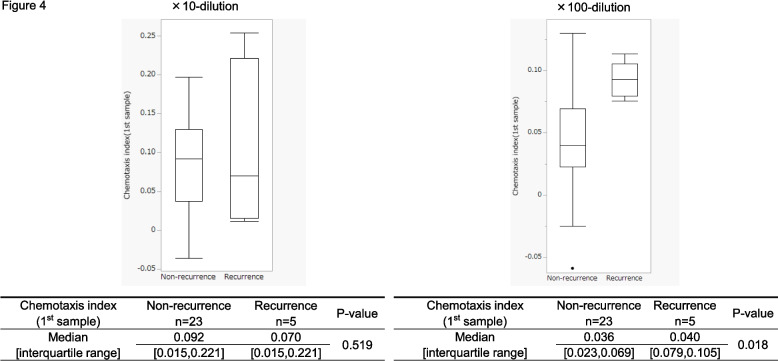
Box plots of chemotaxis responses from recurrence and non-recurrence patients. Analysis using chemotaxis indexes of urine samples of 28 cases with vascular invasion including 5 recurrence and 23 non-recurrence patients showed a significant association of chemotaxis index and recurrence

Next, we focused on the 28 cases with vascular invasion (Supplemental Fig. 1 and 2, diagonal lined bars), which included 5 recurrence and 23 non-recurrence cases. Box plots were created based on the chemotaxis indexes to compare the recurrence and non-recurrence groups (Fig. 4). Before chemotherapy and/or surgery, the chemotaxis indexes (1st sample) of 100-fold diluted urine samples from the 28 patients were significantly greater in the recurrence group than in the non-recurrence group (Fig. 4, *p* = 0.018). In contrast, no significant differences were observed in tenfold diluted urine samples.

**Fig. 5:**
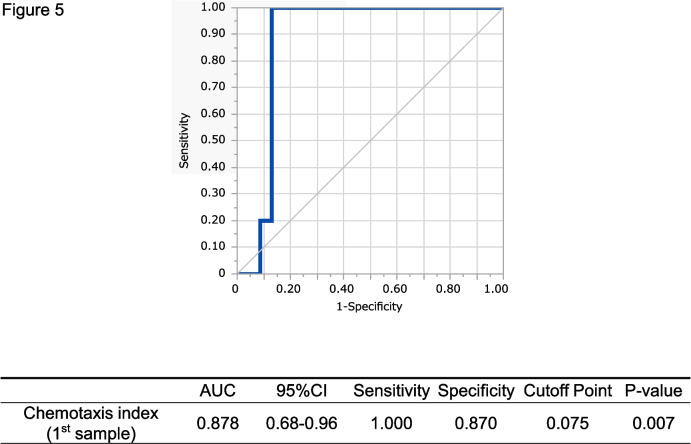
ROC analysis of 28 cases with vascular invasion. The ROC analysis based on chemotaxis index (1st sample) with 100-fold diluted urines of 28 vascular invasion cases showed a high AUC value

ROC analysis of the cases with vascular invasion showed that the cut-off value of N-NOSE indexes at the 1st sample timepoint was 0.075 (Fig. 5). Among tumor markers, only the 1st sample timepoint CEA was significantly greater in the recurrence group (CEA; *p* = 0.039, SCC; *p* = 0.188) (data not shown). Additionally, we investigated whether urinary components affected the chemotactic behavior of the nematode. Urine general qualitative tests demonstrated no statistical association between the N-NOSE index and urinary components such as protein, glucose, or occult blood.

### Follow-up on recurrent cases

**Fig. 6:**
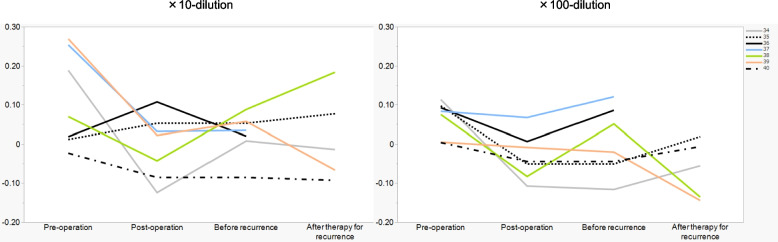
The surveillance follow-up on recurrence cases with N-NOSE. The chemotaxis indexes of seven recurrence cases were monitored at pre-operation, post-operation, before recurrence, and after therapy for recurrence. Five cases, patients #34, 36, 37, 38, and 39 showed similar trends with 10- or 100-fold diluted urine samples, where the positive index of pre-operation samples reduced at post-operation, slightly increased before the recurrence, and three of these patients (#34, 38, and 39) who showed treatment response again dropped after therapy for recurrence

For further investigation, we monitored the chemotaxis indexes in seven recurrent cases in terms of correlation between recurrence and chemotaxis index (Supplemental Fig. 1 and 2, orange bars, patient number 34–40) and plotted the changes in indexes of pre-operation, post-operation, before recurrence, and after recurrence. The sample after recurrence of the patient number 36 is missing due to the diagnosis of recurrence at the end of the 2-year postoperative follow-up period. The sample after recurrence of the patient number 37 is missing due to the transition to best supportive care. Nonetheless, five patients (#34, 36, 37, 38, and 39) showed similar trends with 10- or 100-fold diluted urine samples, where the positive index of pre-operation samples reduced at post-operation, slightly increased before the recurrence was known on imaging, and three of these patients (#34,38 and 39) who showed treatment response again dropped after therapy for recurrence (Fig. 6), indicating a correlation of recurrence and chemotaxis index. These results strongly suggest a potential of N-NOSE as an effective tool for follow-ups on patients with upper gastrointestinal cancer. (#34; pre-operation = 0.188, post-operation = −0.124, before recurrence = 0.008, #36; pre-operation = 0.093, post-operation = 0.006, before recurrence = 0.086, #37; pre-operation = 0.084, post-operation = 0.067, before recurrence = 0.121, #38; pre-operation = 0.075, post-operation = −0.083, before recurrence = 0.051, #39; pre-operation = 0.269, post-operation = 0.022, before recurrence = 0.057).

## Discussion

In this study, we investigated the variability of the N-NOSE index correlation with disease recurrence. Our findings suggest that N-NOSE may serve as a viable biomarker for monitoring patients with vascular invasion. A novel aspect of this study is the longitudinal analysis of the N-NOSE index, tracking the same patients from pre-operation through two years post-operatively, to assess its relationship with cancer recurrence in gastroesophageal malignancies.

The limitations of this study include the small number of patients enrolled and the few cases of recurrence. This implies that subgroup analyses should be interpreted with caution. Additionally, other factors that might have affected the olfactory response of the nematode (e.g., patient nutritional status, medical history, medications) were not sufficiently considered. Further clinical studies with the large numbers of cases with vascular invasion will strengthen the availability of N-NOSE for the surveillance of cancer recurrence.

We investigated the relationship between recurrence and the N-NOSE index. In patients with positive vascular invasion, those who experienced recurrence exhibited statistically significantly higher the 1st sample index values than those without recurrence. Some reports suggest that the risk of recurrence in cases of vascular invasion is not high in esophageal cancer, but there are several recent reports that indicate vascular invasion as a factor in recurrence after surgical treatment [25–29]. Our results suggests that patients with vascular invasion may release tumor-associated substances into the bloodstream and urine, which are detectable by nematodes. These findings indicate that it is possible to identify a high-risk group for recurrence among postoperative patients with vascular invasion using specific cut-off values. Moreover, meticulous monitoring of these patients' postoperative course could facilitate the early detection of recurrence.

In individual recurrence cases, the index decreased after surgery and increased before recurrence. The index decreased again after the start of therapy for recurrence. This suggests an association between the chemotaxis index and therapeutic response. In a previous report, three gastrointestinal cancer patients with recurrence showed a decrease in the N-NOSE index after surgery. The index increased with the recurrence [15]. Our study showed that changes in the chemotaxis index may have occurred even before the recurrence was known on imaging. However, the nematodes showed avoidance behavior after recurrence in two recurrent cases (patient number 35 and 40). In these cases, cancer treatment or physiological changes after surgery may have had an effect on the chemotaxis. Additional research is needed to identify the cause of these nematode-altered reactions.

The study's findings, which show a difference in nematode response at 100-fold urine dilution but not at tenfold dilution, can be reasonably explained by the olfactory response of *C. elegans* and the nature of cancer-related odorants volatile organic compounds (VOCs) in urine. *C. elegans* exhibits non-linear olfactory responses that are highly dependent on odor concentrations. In this study, at 100-fold dilution, the concentration of cancer odorants likely reaches an optimal level for nematode detection, which may allow for differentiation between recurrence and non-recurrence cases. As Yoshida et al*.* demonstrated, the chemotactic response and odor preference of *C. elegans* can easily change: if the concentration of favourite pure odorant is higher than optimal, nematodes show aversive behavior [22]. This aligns with previous research showing peak nematode performance at tenfold and 100-fold urine dilutions. Additionally, the 100-fold dilution may offer a better signal-to-noise ratio by reducing background odors while maintaining sufficient levels of cancer-specific VOCs. This optimal dilution may allow the nematodes to detect and respond to the subtle changes in urinary VOC profiles associated with cancer recurrence, particularly in patients with vascular invasion. Angiogenesis is closely associated with cancer progression and may allow the secreted extracellular cancer-specific VOCs to be circulated via the bloodstream efficiently, possibly leading to the better performance for urine-based cancer detection using nematode olfaction. The consistency of these results with prior studies and the olfactory sensing ability in *C. elegans* further supports the observation of differences at 100-fold but not tenfold dilution, between recurrence and non-recurrence groups. Additionally, post-operative monitoring showed that the chemotaxis index decreased after surgery and increased before recurrence, with this trend appearing to be slightly more at the 100-fold dilution. This indicates that the 100-fold dilution may be reliable for monitoring post-operative changes and detecting early signs of recurrence, potentially serving as a sensitive and specific biomarker for cancer surveillance. These findings are consistent with our previous clinical study, which showed a decreased chemotaxis index following cancer surgery [15].

The fact that nematodes show avoidance behavior after cancer treatment suggests that they react to tumor-associated substances. However, efforts are ongoing to map specific biomarkers and VOCs associated with cancer [30–40]. To translate the findings of this study into a clinically established method for early detection of recurrence, it is crucial to define the conditions under which the tests should be conducted and to determine what results should be considered positive. Follow-up studies should focus on this.

## Conclusion

Our study showed the potential of N-NOSE as a tool for follow-up of upper gastrointestinal cancer patients with vascular invasion. Moreover, the variability in nematode responses to tumor-associated substances suggests that N-NOSE might capture biological changes that other diagnostics miss, potentially optimizing surveillance intervals and improving outcomes through earlier detection and treatment of recurrence.

## Supplementary Information

Supplementary Material 1: Supplemental Figure. 1. Comparison of chemotaxis indexes of 40 cancer cases before and after surgery (100-fold dilution). The C. elegans chemotaxis assays were performed using urine samples collected at before chemotherapy and/or surgery and after chemotherapy, 1-month, 3-month, 6-month, 1-year, and 2-year after surgery from 40 cancer patients. The chemotaxis indexes were calculated based on the chemotaxis assays (100-fold dilution) and aligned in bar graphs with the order from highest to lowest (samples numbered as 1 to 40) at the timepoint of sampling before chemotherapy and/or surgery. Urine samples tested: (A) before chemotherapy and/or surgery, After chemotherapy (B), 1 month (C), 3 months (D), 6 months (E), 1 year (F), and 2 years (G) after surgery. Orange bars: patients with recurrence, and diagonal line bars; patients with vascular invasion, and black arrow heads: patients with chemotherapy. Error bars represented the standard error of the mean.

Supplementary Material 2: Supplemental Figure. 2. Comparison of chemotaxis indexes of 40 cancer cases before and after surgery (tenfold dilution). The C. elegans chemotaxis assays were performed using urine samples collected at before chemotherapy and/or surgery and after chemotherapy, 1-month, 3-month, 6-month, 1-year, and 2-year after surgery from 40 cancer patients. The chemotaxis indexes were calculated based on the chemotaxis assays (tenfold dilution) and aligned in bar graphs with samples numbered as 1 to 40 at the timepoint of sampling. Urine samples tested: (A) before chemotherapy and/or surgery, After chemotherapy (B), 1 month (C), 3 months (D), 6 months (E), 1 year (F), and 2 years (G) after surgery. Orange bars: patients with recurrence, and diagonal line bars; patients with vascular invasion, and black arrow heads: patients with chemotherapy. Error bars represented the standard error of the mean.

## Data Availability

The datasets used and analyzed during the current study are available from the corresponding author upon reasonable request.

## Abbreviations

N-NOSE: Nematode-NOSE
ROC: Receiver operating characteristic
AUC: Area under the curve
CEA: Carcinoembryonic antigen
CA19-9: Carbohydrate antigen 19–9
SCC: Squamous cell carcinoma antigen
*C. elegans*: *Caenorhabditis elegans*
ECOG: Eastern Cooperative Oncology Group
PS: Performance status
DCF: Docetaxel, cisplatin, 5-FU
CT: Computed tomography
RECIST: Response Evaluation Criteria in Solid Tumors Criteria
PET-CT: Positron emission tomography-computed tomography
NGM: Nematode Growth Media
*E. coli*: *Escherichia coli*
CR: Complete response
PR: Partial response
SD: Stable disease
PD: Progressive disease
VOC: Volatile Organic Compound
