# Correction: Evaluation of N-NOSE as a surveillance tool for recurrence in gastric and esophageal cancers: a prospective cohort study

**DOI:** 10.1186/s12885-025-13451-2

**Published:** 2025-01-09

**Authors:** Sayuri Iitaka, Akihiro Kuroda, Tomonori Narita, Hideyuki Hatakeyama, Masayo Morishita, Umbhorn Ungkulpasvich, Takaaki Hirotsu, Eric di Luccio, Koichi Yagi, Yasuyuki Seto

**Affiliations:** 1 Department of Gastrointestinal Surgery, Graduate School of Medicine, The University of Tokyo, Tokyo, 113-8655 Japan; 2 Hirotsu Bio Science Inc, Tokyo, 102-0094 Japan; 3 Present address: National Cancer Center Hospital, Tokyo, 104-0045 Japan

**Correction: BMC Cancer 24, 1544 (2024)**

**https://doi.org/10.1186/s12885-024–13327-x**

Following publication of the original article [1], it was reported that the corresponding author was incorrectly marked. Yasuyuki Seto is the corresponding author.

The original article [1] has been corrected.
