# Supplementary material for: Evaluation of N-NOSE as a surveillance tool for recurrence in gastric and esophageal cancers: a prospective cohort study
Source: BMC Cancer. 2024 Dec 18;24:1544. doi: 10.1186/s12885-024-13327-x (PMC11656990; doi:10.1186/s12885-024-13327-x)
Supplement: Supplementary file 1 — Supplementary Material 1: Supplemental Figure. 1. Comparison of chemotaxis indexes of 40 cancer cases before and after surgery (100-fold dilution). The C. elegans chemotaxis assays were performed using urine samples collected at before chemotherapy and/or surgery and after chemotherapy, 1-month, 3-month, 6-month, 1-year, and 2-year after surgery from 40 cancer patients. The chemotaxis indexes were calculated based on the chemotaxis assays (100-fold dilution) and aligned in bar graphs with the order from highest to lowest (samples numbered as 1 to 40) at the timepoint of sampling before chemotherapy and/or surgery. Urine samples tested: (A) before chemotherapy and/or surgery, After chemotherapy (B), 1 month (C), 3 months (D), 6 months (E), 1 year (F), and 2 years (G) after surgery. Orange bars: patients with recurrence, and diagonal line bars; patients with vascular invasion, and black arrow heads: patients with chemotherapy. Error bars represented the standard error of the mean. [file 12885_2024_13327_MOESM1_ESM.pdf]

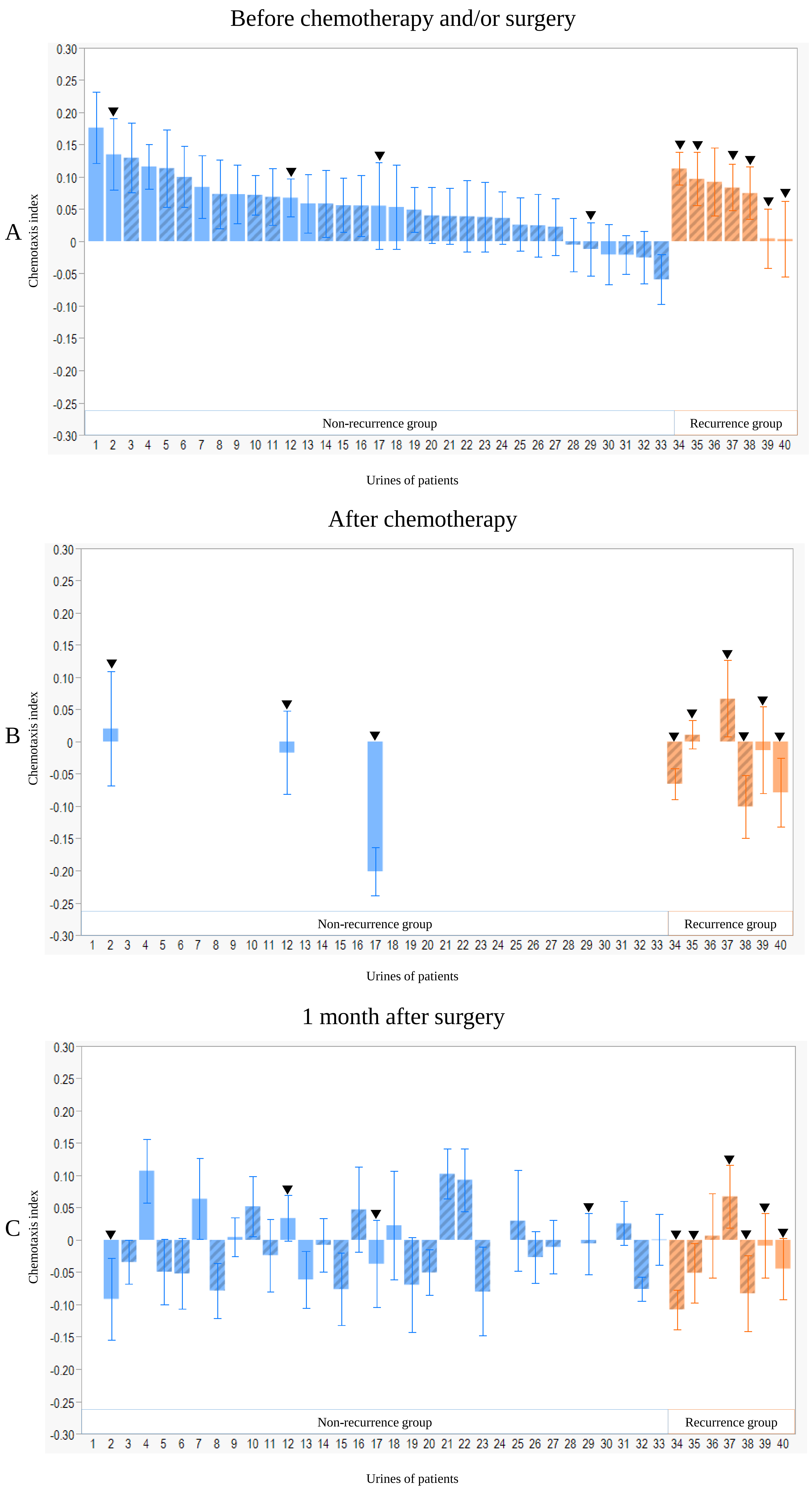
Before chemotherapy and/or surgery
A
Chemotaxis index
0.30
0.25
0.20
0.15
0.10
0.05
0
-0.05
-0.10
-0.15
-0.20
-0.25
-0.30
Non-recurrence group
Recurrence group
1 2 3 4 5 6 7 8 9 10 11 12 13 14 15 16 17 18 19 20 21 22 23 24 25 26 27 28 29 30 31 32 33 34 35 36 37 38 39 40
Urines of patients
After chemotherapy
B
Chemotaxis index
Non-recurrence group
Recurrence group
Urines of patients
1 month after surgery
C
Chemotaxis index
Non-recurrence group
Recurrence group
Urines of patients

3 months after surgery

D

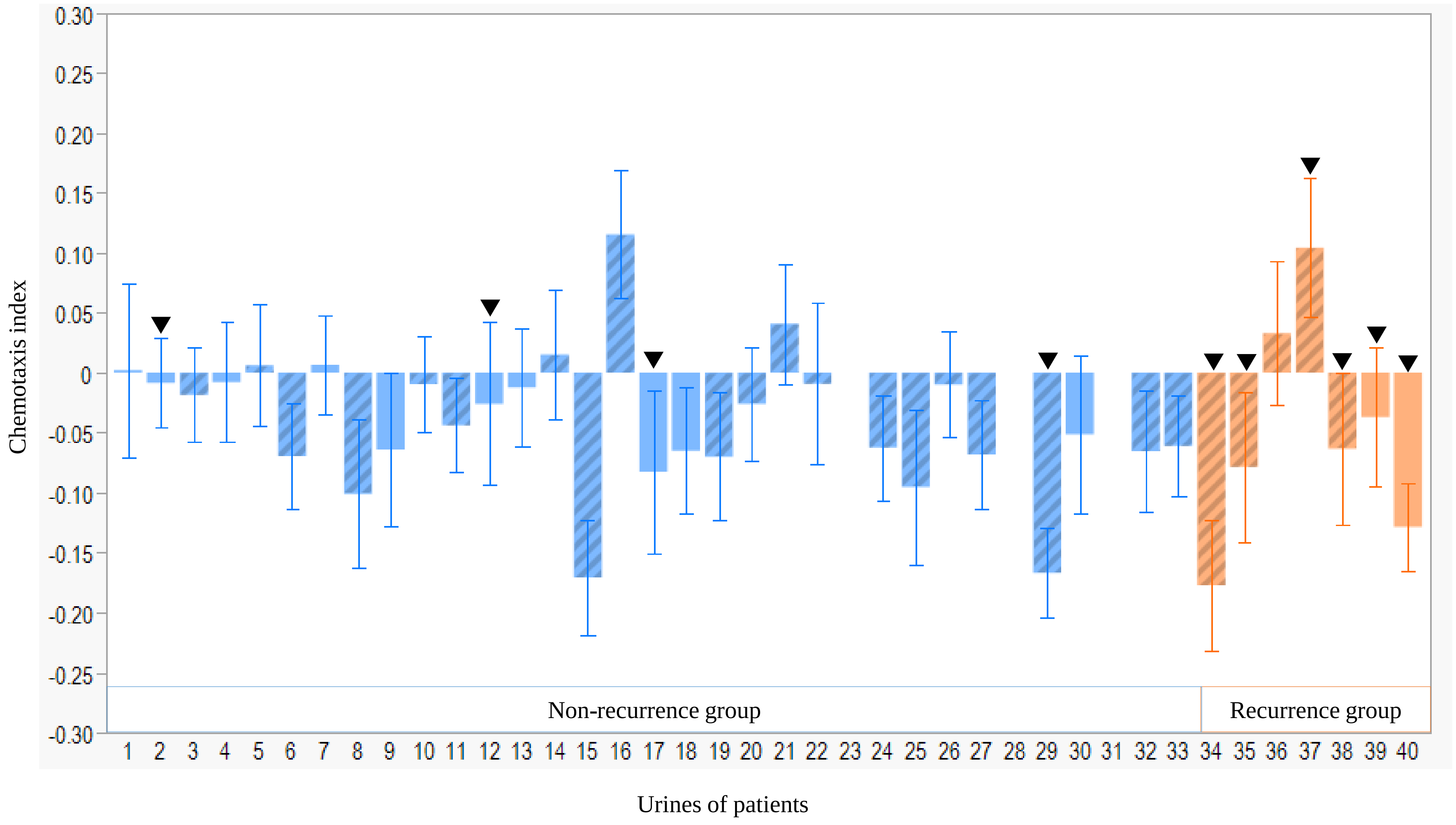

6 months after surgery

E

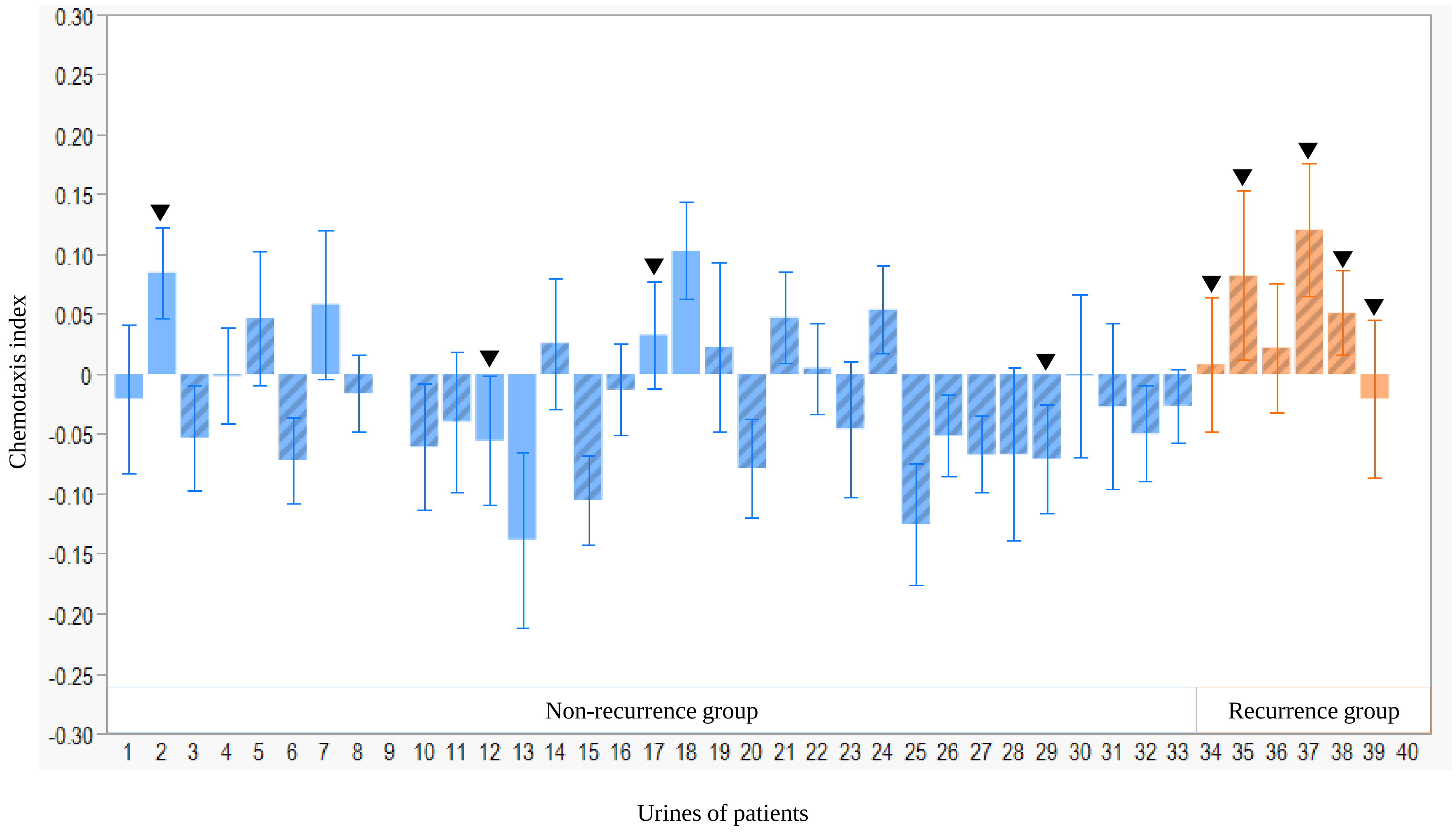

1 year after surgery

F

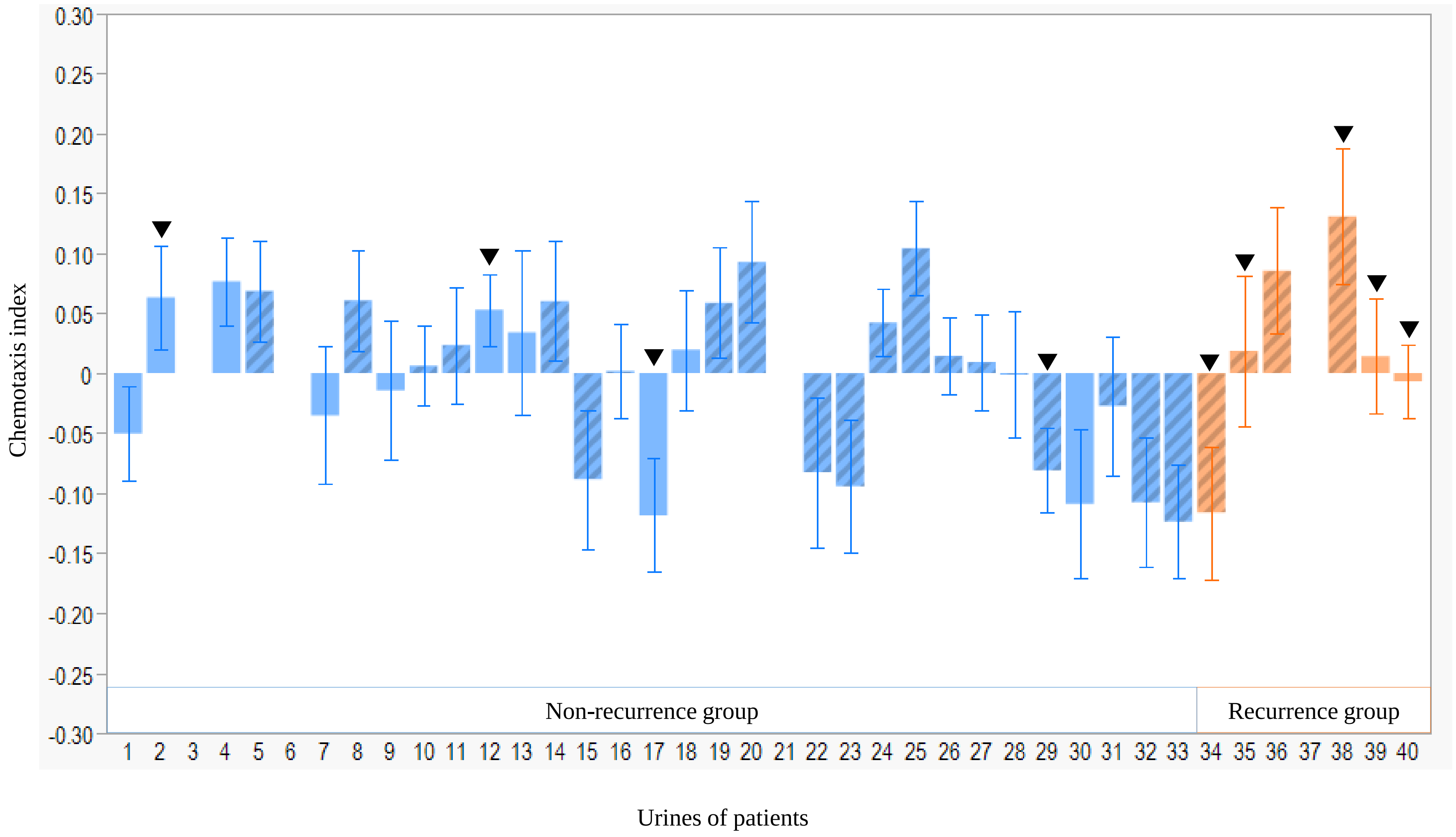

2 years after surgery

G

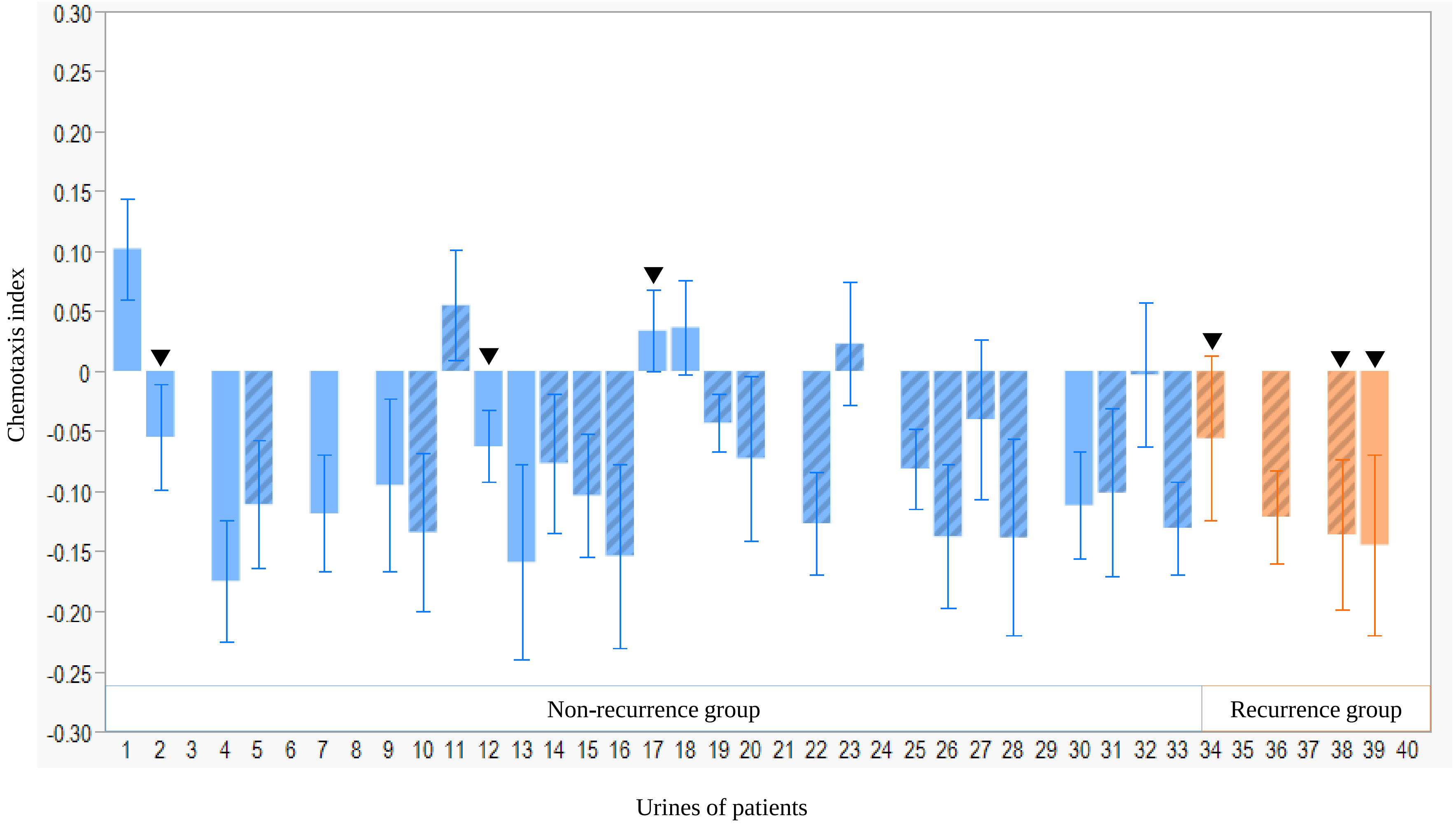

Supplemental Figure 1. Comparison of chemotaxis indexes of 40 cancer cases before and after surgery (100-fold dilution). The C. elegans chemotaxis assays were performed using urine samples collected at before chemotherapy and/or surgery and after chemotherapy, 1-month, 3-month, 6-month, 1-year, and 2-year after surgery from 40 cancer patients. The chemotaxis indexes were calculated based on the chemotaxis assays (100-fold dilution) and aligned in bar graphs with the order from highest to lowest (samples numbered as 1 to 40) at the timepoint of sampling before chemotherapy and/or surgery. Urine samples tested: (A) before chemotherapy and/or surgery, After chemotherapy (B), 1 month (C), 3 months (D), 6 months (E), 1 year (F), and 2 years (G) after surgery. Orange bars: patients with recurrence, and diagonal line bars; patients with vascular invasion, and black arrow heads: patients with chemotherapy. Error bars represented the standard error of the mean.
