# Supplementary material for: Evaluation of N-NOSE as a surveillance tool for recurrence in gastric and esophageal cancers: a prospective cohort study
Source: BMC Cancer. 2024 Dec 18;24:1544. doi: 10.1186/s12885-024-13327-x (PMC11656990; doi:10.1186/s12885-024-13327-x)
Supplement: Supplementary file 2 — Supplementary Material 2: Supplemental Figure. 2. Comparison of chemotaxis indexes of 40 cancer cases before and after surgery (tenfold dilution). The C. elegans chemotaxis assays were performed using urine samples collected at before chemotherapy and/or surgery and after chemotherapy, 1-month, 3-month, 6-month, 1-year, and 2-year after surgery from 40 cancer patients. The chemotaxis indexes were calculated based on the chemotaxis assays (tenfold dilution) and aligned in bar graphs with samples numbered as 1 to 40 at the timepoint of sampling. Urine samples tested: (A) before chemotherapy and/or surgery, After chemotherapy (B), 1 month (C), 3 months (D), 6 months (E), 1 year (F), and 2 years (G) after surgery. Orange bars: patients with recurrence, and diagonal line bars; patients with vascular invasion, and black arrow heads: patients with chemotherapy. Error bars represented the standard error of the mean. [file 12885_2024_13327_MOESM2_ESM.pdf]

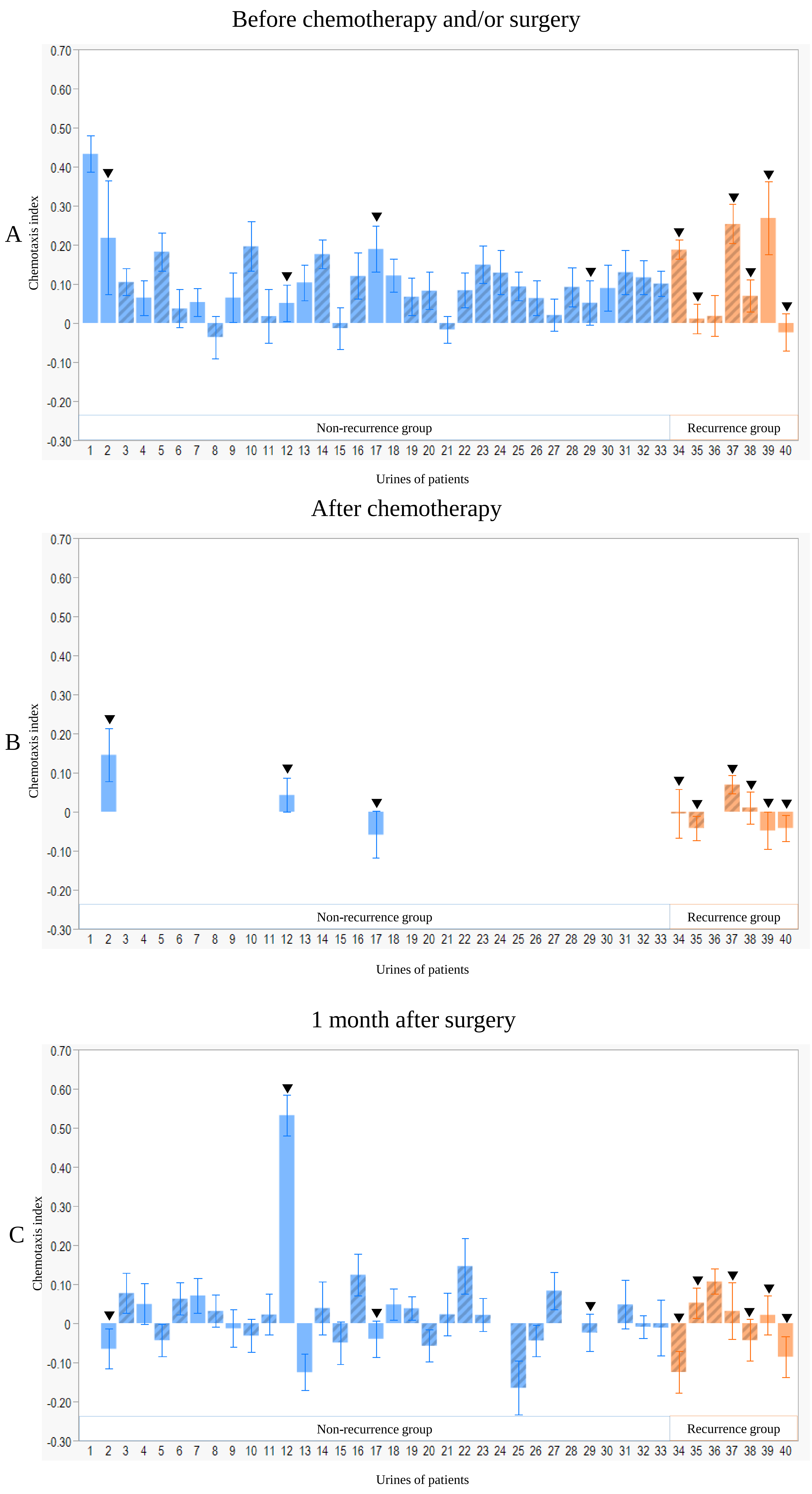

Before chemotherapy and/or surgery
A
Chemotaxis index
0.70
0.60
0.50
0.40
0.30
0.20
0.10
0
-0.10
-0.20
-0.30
Non-recurrence group
Recurrence group
1 2 3 4 5 6 7 8 9 10 11 12 13 14 15 16 17 18 19 20 21 22 23 24 25 26 27 28 29 30 31 32 33 34 35 36 37 38 39 40
Urines of patients
After chemotherapy
B
Chemotaxis index
0.70
0.60
0.50
0.40
0.30
0.20
0.10
0
-0.10
-0.20
-0.30
Non-recurrence group
Recurrence group
1 2 3 4 5 6 7 8 9 10 11 12 13 14 15 16 17 18 19 20 21 22 23 24 25 26 27 28 29 30 31 32 33 34 35 36 37 38 39 40
Urines of patients
1 month after surgery
C
Chemotaxis index
0.70
0.60
0.50
0.40
0.30
0.20
0.10
0
-0.10
-0.20
-0.30
Non-recurrence group
Recurrence group
1 2 3 4 5 6 7 8 9 10 11 12 13 14 15 16 17 18 19 20 21 22 23 24 25 26 27 28 29 30 31 32 33 34 35 36 37 38 39 40
Urines of patients

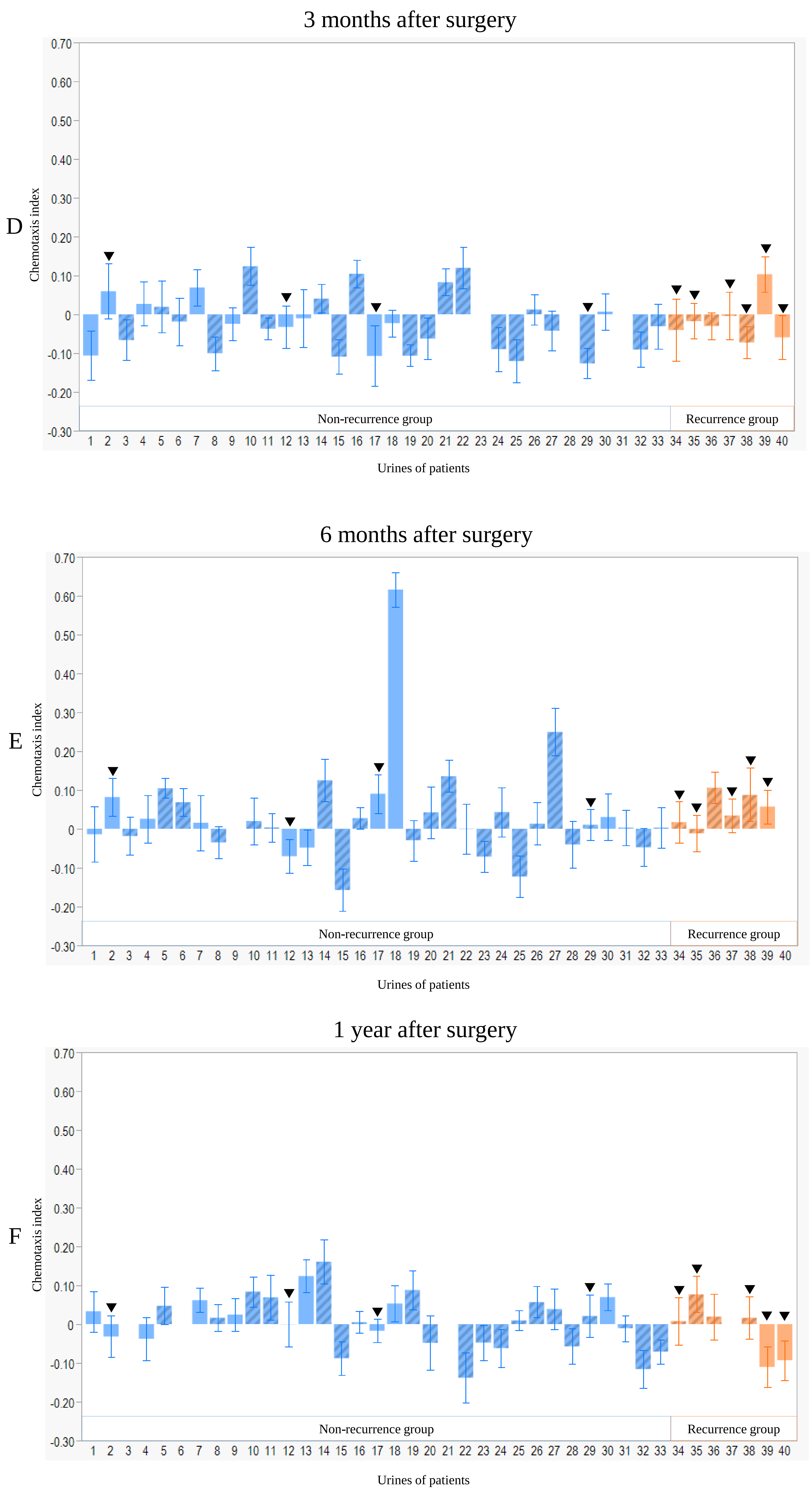
3 months after surgery
D
Chemotaxis index
Non-recurrence group
Recurrence group
Urines of patients
6 months after surgery
E
Chemotaxis index
Non-recurrence group
Recurrence group
Urines of patients
1 year after surgery
F
Chemotaxis index
Non-recurrence group
Recurrence group
Urines of patients

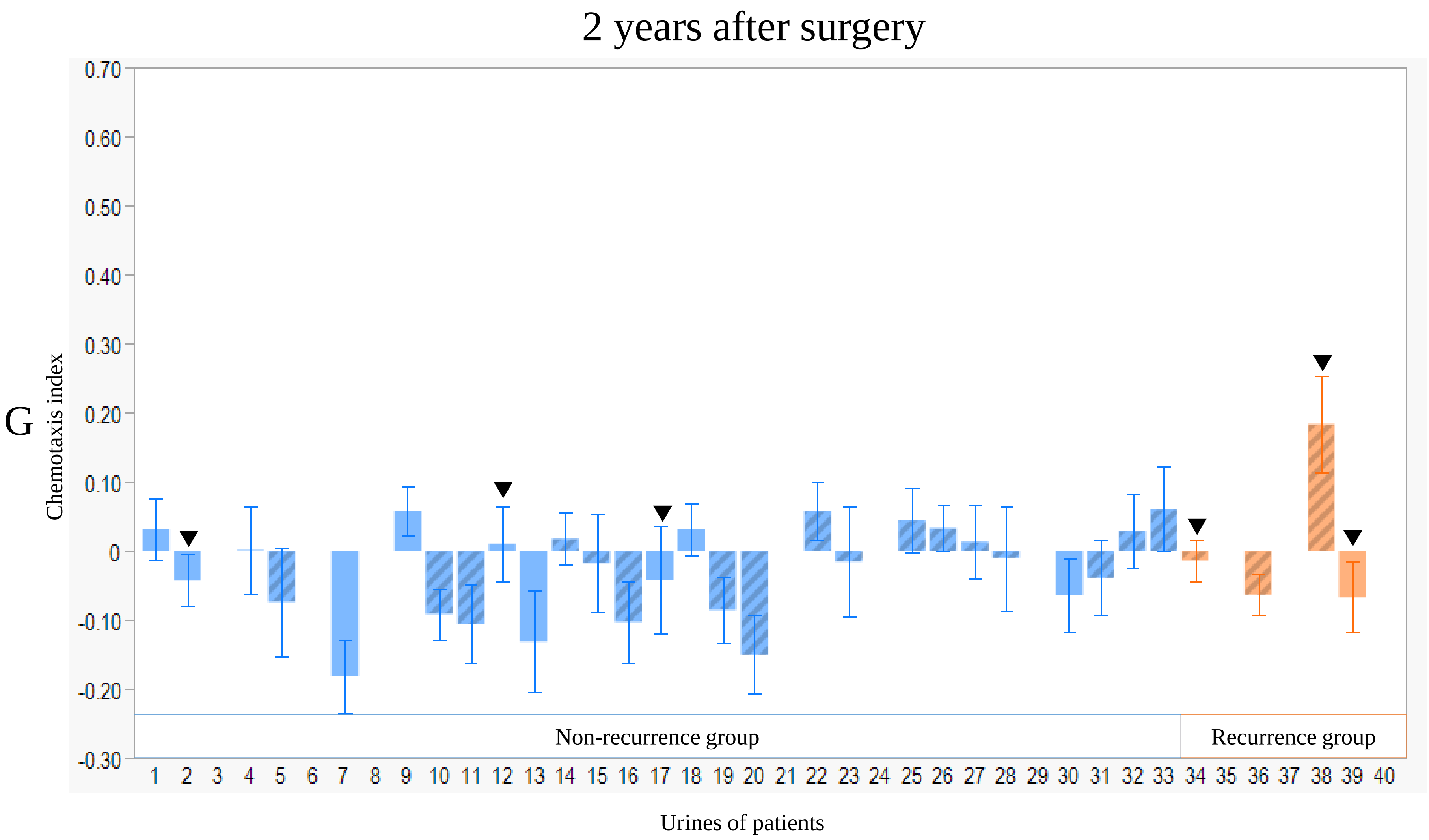

Supplemental Figure 2. Comparison of chemotaxis indexes of 40 cancer cases before and after surgery (10-fold dilution). The *C. elegans* chemotaxis assays were performed using urine samples collected at before chemotherapy and/or surgery and after chemotherapy, 1-month, 3-month, 6-month, 1-year, and 2-year after surgery from 40 cancer patients. The chemotaxis indexes were calculated based on the chemotaxis assays (10-fold dilution) and aligned in bar graphs with samples numbered as 1 to 40 at the timepoint of sampling. Urine samples tested: (A) before chemotherapy and/or surgery, After chemotherapy (B), 1 month (C), 3 months (D), 6 months (E), 1 year (F), and 2 years (G) after surgery. Orange bars: patients with recurrence, and diagonal line bars; patients with vascular invasion, and black arrow heads: patients with chemotherapy. Error bars represented the standard error of the mean.
